# Expectations for the 2020 Decennial Census and How They Stood Up to Scrutiny.

**DOI:** 10.23889/ijpds.v7i3.2030

**Published:** 2022-08-25

**Authors:** Isabel Youngs, Christopher Dick, Ronald Prevost, Claire Bowen

**Affiliations:** 1 Curtin University; 2 Urban Institute

The importance of the decennial census is clear, but the 2020 Census faced unprecedented challenges with unknown effects on data quality. To assist data users in identifying deviations between expected counts and the released counts across population and housing indicators, we developed the 2020 Census County Assessment Tool. The tool offers contextual data for each county in the US on factors which could have contributed to census collection issues, including COVID-19, internet-first response, natural disasters, and broadband access rates. The tool also allows users to quickly see the distributions of divergence across race, ethnicity, and housing, allowing a user to dive into the data for their specific use. The tool compiles this information into downloadable reports and points users to additional local data sources or experts to seek more assistance.

This tool assists planners, administrators, policymakers, teachers and students, reporters, researchers, and anyone interested in census data in identifying strategies for utilizing census data appropriately. This tool particularly provides resources to data users interested in the accurate enumeration of race and ethnicity, pointing users to areas which may need additional attention for the 2030 census. This tool can assist users in intercensal planning, count review requests, population estimates challenges, funding allocation corrections, Get Out the Count investment strategies, and more. This presentation will discuss the development process and methodology of the tool, as well as our plans for future additions and other tools we are in the process of creating.

